# Properties of 3-Dimensional Cell Cultivation Matrices and Scaffolds in Magnetic Resonance Imaging at 3 Tesla

**DOI:** 10.3390/jfb16120440

**Published:** 2025-11-26

**Authors:** Rebecca Wißmann, Petros Martirosian, Marina Danalache, Stefanie Elser, Jürgen Machann, Fritz Schick

**Affiliations:** 1 Department of Diagnostic and Interventional Radiology, University Hospital of Tübingen, D-72076 Tübingen, Germanyjuergen.machann@med.uni-tuebingen.de (J.M.);; 2 Section on Experimental Radiology, Department of Diagnostic and Interventional Radiology, University Hospital of Tübingen, D-72076 Tübingen, Germany; 3 Department of Orthopedic Surgery, University Hospital of Tübingen, D-72076 Tübingen, Germany; 4 Institute for Diabetes Research and Metabolic Diseases, Helmholtz Munich at the University of Tübingen, D-72076 Tübingen, Germany; 5 German Center for Diabetes Research (DZD), D-72076 Tübingen, Germany

**Keywords:** matrices, scaffolds, spheroids, magnetic resonance imaging (MRI), proton magnetic resonance spectroscopy (^1^H-MRS), 3D culture, tumor model, regenerative medicine, tissue engineering

## Abstract

Natural or synthetic scaffolds are essential for developing three-dimensional (3D) cell culture models, as they provide structural stability and accurately replicate the cellular microenvironment. When integrated into optimized setups, scaffold-supported cellular aggregates, such as spheroids, can be non-destructively characterized and monitored using 3T Magnetic Resonance Imaging (MRI). However, a significant technical limitation is the presence of MR artifacts generated by scaffolds, which can severely obscure the visualization of the embedded spheroids. This study systematically evaluated the suitability of various scaffolds and matrices (including Matrigel^®^, fibrin glue, and several hydrogels) for MRI and MR spectroscopy (MRS). The materials were investigated both native and seeded with chondrosarcoma cells (SW1353). Our findings revealed considerable variability in MR compatibility across different materials. Specifically, fibrin glue proved unsuitable for MR applications due to substantial artifact generation that interfered with the visualization of cellular components. Furthermore, the results emphasize the importance of the observation period, as material degradation processes can introduce confounding factors in longitudinal MR studies. The choice of scaffold material is paramount for the successful analysis of 3D cell models via MRI. Careful selection is required, as the materials’ properties and temporal stability directly impact the interpretability of the acquired data.

## 1. Introduction

Three-dimensional (3D) cell culture systems have gained significant traction in preclinical research due to their ability to more accurately replicate the physiological conditions of the in vivo cellular microenvironment. Unlike traditional two-dimensional (2D) cultures, which often fail to recapitulate the complexity of cell–cell and cell–matrix interactions, 3D systems have been shown to profoundly influence cellular behaviors, including proliferation, differentiation, gene expression, and metabolic function [1,2,3]. This shift towards 3D models stems from the growing recognition that cellular responses in 2D may not translate effectively to in vivo settings [4]. Despite the advantages of 3D cultures, not all cell types possess the inherent capacity to self-assemble into three-dimensional architectures. In such cases, structural support is essential to facilitate spatial organization and functional integration.

These scaffolds serve as a surrogate extracellular matrix (ECM), directing cell attachment, migration, proliferation, and differentiation. Solid scaffolds provide several advantages, such as the capacity for customization to direct cell patterning and the controlled presentation of biochemical signals with temporal and spatial precision [5,6]. The benefits of scaffold-based systems have been well-documented across a range of applications. Enhanced cell viability, function, and differentiation have been demonstrated in engineered models of bone [7], skin [8], cancer [9], cardiac [10], and skeletal muscle tissue [11]. Scaffolds are composed of 3D porous, fibrous, or permeable biomaterials and can be derived from natural sources, or made from synthetic polymers that may be engineered to mimic the biological activities of the ECM [12,13].

Among naturally derived matrices, Matrigel^®^ remains one of the most widely used scaffolds for 3D cell culture. Matrigel^®^ consists of the isolated basement membrane of the Elbrecht–Holm–Swarm (EHS) tumor, a chondrosarcoma line that occurs in mice. Matrigel^®^ contains the main components of the extracellular matrix, such as collagen, laminin, and proteoglycans, and forms a clear stable gel through aggregation of proteins when heated to 37 °C [14]. The use of Matrigel^®^ for various applications in tissue engineering has been studied in over 12,000 publications with a great variety of cell lines [15]. It has been shown that cell cultivation on Matrigel^®^ can enhance proliferation [16,17] and even promote cell differentiation [18,19,20]. The natural origin of Matrigel^®^ also causes some limitations; the lack of high proportions of collagen I and hyaluronic acid in Matrigel^®^, for example, does not correspond to their high proportions in in vivo tumor microenvironments and their importance to structure and tissue architecture [21]. Furthermore, the presence and abundance of other components such as hormones and growth factors are unknown and can therefore result in uncontrolled effects on cancer cell behavior [22]. To overcome such limitations, synthetic hydrogels have gained attention as versatile and highly tunable alternatives.

Fibrin glue (FG) represents another naturally derived scaffold that mimics physiological tissue repair processes. Due to its natural compounds, FG is considered non-toxic, non-allergenic, and biodegradable. FG acts as a cell-delivery vehicle, and facilitates cell attachment, proliferation, differentiation, and ultimately, tissue formation and organization due to its three-dimensional cell structure [23,24].

Furthermore, biologically relevant polymers such as hyaluronic acid (HA), a key glycosaminoglycan in native ECM, have been widely integrated into synthetic hydrogels. HA plays essential roles in cellular signaling, morphogenesis, wound healing, and matrix remodeling [25,26]. Its chemical structure allows for extensive modification, enabling the creation of hydrogels with tunable stiffness, bioactivity, and degradation rates [27,28]. HA-based hydrogels are already in clinical use as dermal fillers, viscosupplements, and wound dressings, reflecting their translational potential [29,30].

Hydrogels derived from cross-linked polyethylene glycol (PEG) have versatile areas of application. The polymer is biologically inert and therefore non-toxic and non-immunogenic, providing an ideal base for a synthetic scaffold. PEG has an active hydroxyl end, so it can be combined with various bioactive molecules such as drugs, cytokines, and growth factors [31].

In addition to hydrogel-based systems, polystyrene (PS)-based 3D inserts offer a fully synthetic scaffold alternative. These scaffolds, typically fabricated via additive manufacturing techniques, present a rigid, porous architecture that supports cell adhesion and the formation of multicellular spheroids [32]. Owing to their chemical inertness, PS-based inserts offer a versatile platform for the modular incorporation of ECM-mimetic components, enabling precise customization of the cellular microenvironment [33].

Given the increasing reliance on 3D culture systems for functional tissue modeling, there is a growing need to assess their compatibility with non-invasive imaging modalities such as magnetic resonance imaging (MRI) and magnetic resonance spectroscopy (MRS). MRI enables detailed, voxel-level mapping of structural and functional parameters, including T1 and T2 relaxation times, apparent diffusion coefficients (ADCs), and magnetization transfer ratios (MTRs). These parameters reflect tissue characteristics such as water mobility, macromolecular density, and ion content [34].

Complementing these structural insights, ^1^H-MRS provides metabolic profiling by detecting signals from biomolecules such as lipids, amino acids, and choline derivatives [35]. However, both MRI and MRS techniques are highly sensitive to magnetic field inhomogeneity [36]. Even trace amounts of paramagnetic or ferromagnetic materials in scaffold compositions can introduce significant susceptibility artifacts, leading to signal loss, spatial distortion, or spectral overlap [37]. This is particularly problematic for spectroscopy, where unresolved or broadened peaks may obscure metabolite identification and quantification. Since many scaffold formulations and additives are not fully characterized with respect to their magnetic properties, their influence on MR imaging must be evaluated experimentally.

In the present study, we investigate the suitability of various natural and synthetic scaffold materials for MR-based imaging of 3D spheroid cultures using high-field (3 T) MRI and MRS on whole-body MRI systems. We systematically assess the physicochemical characteristics, biological compatibility, and MR imaging artifacts of each scaffold to determine their potential for use in integrated imaging and tissue engineering applications.

## 2. Materials and Methods

### 2.1. Cell Culture and Spheroid Generation

Human SW1353 chondrosarcoma cells (American Type Culture Collection (ATCC), Manassas, VA, USA, HTB-94) were cultivated in DMEM: Ham’s F12 (Gibco/Thermofisher, Waltham, MA, USA) + 10% (*v*/*v*) FCS + 1% (*v*/*v*) penicillin–streptomycin at 37 °C in an incubator containing 5% CO_2_. At an approximate confluence of 80%, cells were detached and counted with a cell counter. Cells were seeded into low-attachment 96-well plates (Thermo Scientific, Waltham, MA, USA) at densities of 62,500 cells per well and incubated for five days under standard cell culture conditions (37 °C, 5% CO_2_, and normoxia).

### 2.2. Scaffolds Preparation

#### Matrigel^®^

Phenol-Red-free Matrigel^®^ (Corning Incorporated, New York, NY, USA) was thawed under sterile conditions on ice and was divided into 2 mL aliquots, which were stored at −20 °C. For each experiment, an aliquot was slowly thawed on ice, mixed, and transferred into a 50 mL Falcon Tube (Corning Incorporated, NY, USA). To solidify the gel, the tube was placed into 37 °C water bath for approximately 20 min. The spheroids were placed in the warmed up Matrigel^®^ after 10 min and then cured for another 10 min at 37 °C.

### 2.3. Hydrogels

Hydrogels (3-D Life PVA PEG Hydrogel SG, 3-D Life Dextran-PEG Hydrogel SG, 3-D Life PVA-HA Hydrogel, Cellendes GmbH, Reutlingen, Germany) were prepared according to manufacturer’s instructions aiming for a final concentration of 2 mmol/L of thiol groups and thiol-reactive groups. A final volume of 2 mL was transferred into a 50 mL Falcon Tube followed by the application of spheroids. The gels underwent a hardening process, following which they were covered with a warm medium.

### 2.4. Fibrin Glue

TISSEEL Fibrin Sealant (Baxter, Deerfield, IL, USA) was supplied as a kit consisting of two prefilled syringes that contained a sealer protein solution (67–106 mg/mL fibrinogen, 2250–3750 IU/mL synthetic aprotinin, and a solution of 400–625 IU/mL thrombin (human)). The sealant was mixed and hardened when applying the contents of the two-compartment syringe on 1% agar. Initially, the mold was lined with fibrin glue, after which the spheroids were added. The residual fibrin glue was subsequently administered to the cell mass, ensuring comprehensive coverage of the spheroids.

### 2.5. Polystyrene Insert

Polystyrene insert (3D Biotek, LLC, North Brunswick Township, NJ, USA) was placed under sterile conditions in a 50 mL Falcon tube on 20 mL 1% agar. Spheroids were subsequently added and placed on the scaffold.

### 2.6. Magnetic Resonance Imaging and Volume Localized Spectroscopy Techniques

#### MRI

**Figure 1:**
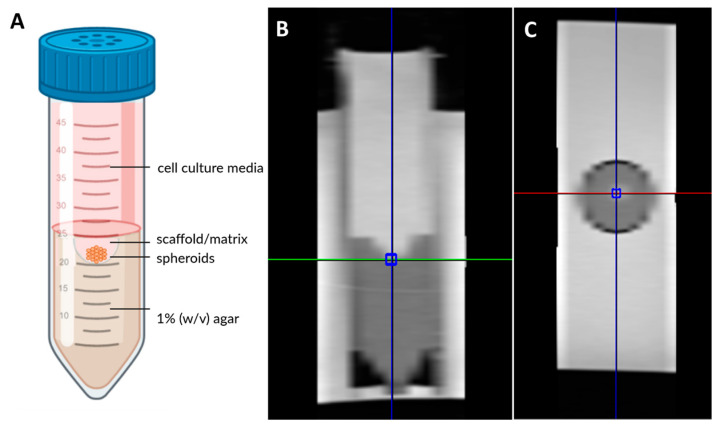
Schematic overview of the setup, designed using Biorender.com. (**A**) Sagittal and (**B**) cross-sectional (**C**) image of measurement positioning for spectroscopy.

All measurements were performed on a whole-body 3T MRI scanner (MAGNETOM Prisma^Fit^, Siemens Healthcare, Erlangen, Germany). Three cultivation tubes (Figure 1) were placed into a plastic box filled with room-tempered (21 °C) double-distilled H_2_O, which was secured into the head coil of the manufacturer [38]. The measurement protocol included a series of sequences for structural and parametric imaging. Proton-density (PD)- and T2-weighted 2D turbo spin echo (TSE) and T1-weighted 3D spoiled gradient echo (VIBE) sequences were employed for high-resolution structural imaging. For quantitative characterization by precise T1 mapping, a 3D variable flip angle (VFA) approach utilizing a VIBE sequence and a preceding B1-correction scan was employed. A two-dimensional Carr–Purcell–Meiboom–Gill (CPMG) multiple spin echo sequence was used for T2 mapping. Magnetization transfer ratio (MTR) was determined through the use of a 3D spoiled gradient echo (GRE) sequence. A diffusion-weighted readout-segmented echo planar imaging (DWI-EPI) technique with b-values of 0, 50, 500, and 1000 s/mm^2^ and a monopolar diffusion gradient scheme was employed to quantify the apparent diffusion coefficient (ADC) for assessment of the microscopic mobility of water molecules. The overall image acquisition took approx. 36 min. A summary of the imaging protocol is provided in Table 1, and all measurements were performed three times independently with different samples (after identical preparation procedures). In order to ensure the quality of the images, signal-to-noise ratio (SNR) was calculated for proton-density-weighted images according to S/s with S is the mean signal intensity of a region of interest in the area of scaffold or matrix and s is the standard deviation of the noise in the image background. In addition, the contrast between the spheroids and matrices in the different imaging modalities was calculated as follows, where S_1_ and S_2_ are signal intensities of matrices and spheroids, respectively:

$$\frac{S_{2}-S_{1}}{S_{2}}\times 100$$

### 2.7. MR Spectroscopy

To perform localized MRS analysis, a short-echo time Stimulated Echo Acquisition Mode (STEAM) sequence was employed. The acquisition parameters were optimized to ensure high spectral quality and adequate signal-to-noise ratio while preserving metabolite detectability. The full set of MRS acquisition parameters used in the experimental approach is summarized in Table 2.

### 2.8. Quantification and Statistical Data Analysis

The images recorded and derived parameter maps were displayed using a self-written MATLAB code (R2024A, The Mathworks Inc., Natick, MA, USA). The mapping parameters of the spheroids were determined by defining regions of interest (ROIs) and analyzed in three independent measurements in our exemplary studies. All statistical and graphical analyses were conducted using GraphPad Prism, version 10.1.1 (GraphPad Software Inc., San Diego, CA, USA). Data are reported as mean ± standard deviation (SD).

## 3. Results

The positioning of the matrices in our configuration was initially established through a qualitative analysis of T1- and T2-weighted images. The SNR in the proton density measurement was determined in order to establish whether the matrices emit signals, thus enabling the initial testing of their general suitability for quantitative MRI evaluation (Table 3). In view of the fact that the imaging slice thickness exceeded the thickness of the polystyrene insert, a quantitative evaluation was not conducted in this instance.

### 3.1. Matrigel^®^

**Figure 2:**
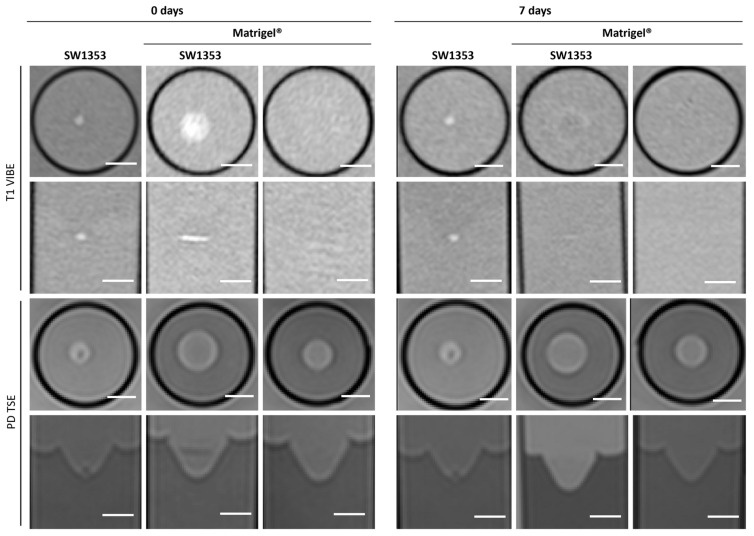
Cross-sectional (1st and 3rd row) and sagittal (2nd and 4th row) images of SW1353 spheroids without and with Matrigel^®^ and just Matrigel^®^. T1-weighted images (1st and 2nd row were obtained using a spoiled gradient echo VIBE sequence. PD-weighted images were obtained using a TSE sequence. Scale bars represent 10 mm.

**Figure 3:**
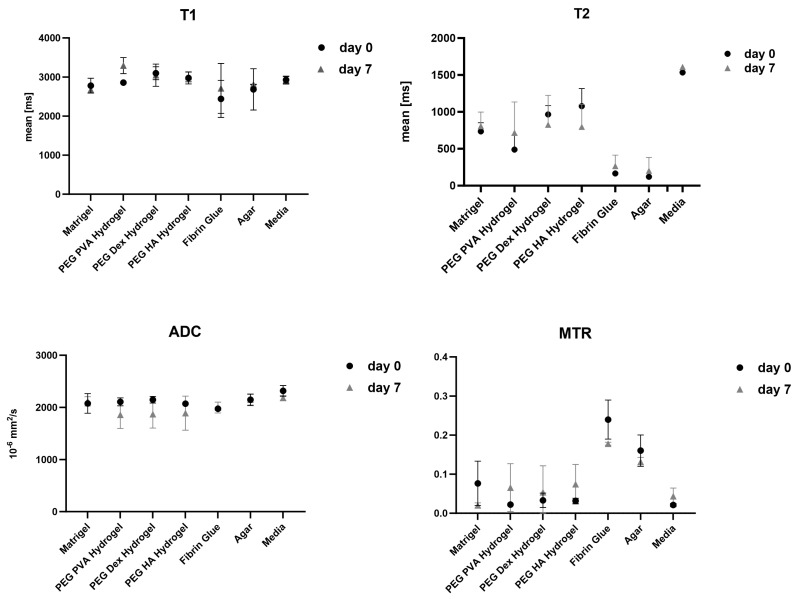
Graphical representation of T1, T2, apparent diffusion coefficient (ADC), and magnetiza-tion transfer ratio (MTR) values measured across different scaffold materials and cell culture media at different time points (day 0 and 7). Data are presented as mean ± standard deviation from three independent experiments. Each parameter reflects quantitative MR-based assessment of scaffold-specific properties under identical imaging conditions.

**Figure 4:**
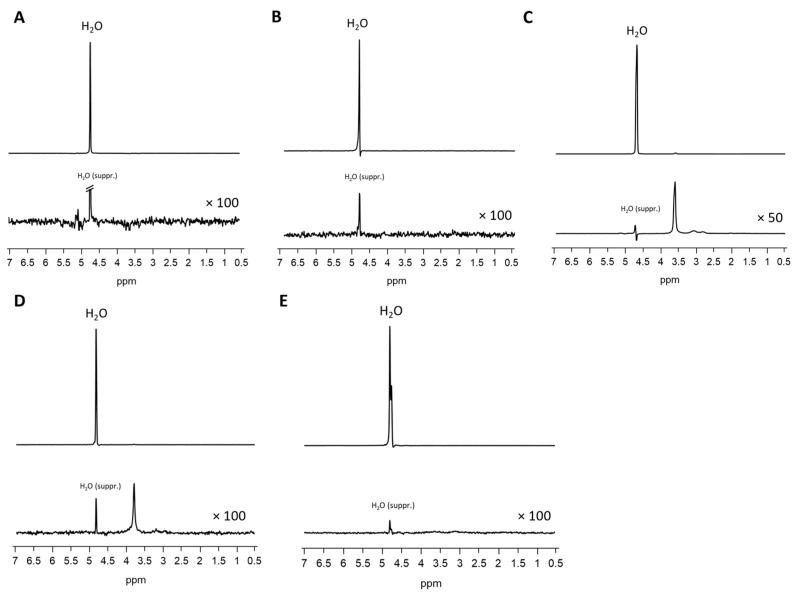
MRS of Matrigel^®^ (**A**), fibrin glue (**B**), PEG PVA hydrogel (**C**), PEG Dex hydrogel (**D**), and HA PVA hydrogel (**E**). Upper lines: spectra without water suppression, lower rows: zoomed spectra with water suppression.

In T1-weighted imaging, Matrigel^®^ cannot be distinguished from the surrounding agar or medium (Figure 2). PD-weighted imaging allows for contrast with the agar, but localization of the Matrigel^®^ is challenging. The signal from the spheroids stands out from the surrounding matrix, especially in T1-weighted imaging with the contrast dropping from 30% to 3% (Table 3). However, this signal is significantly attenuated after 7 days in both image modalities, thus hindering the localization of cell spheroid clusters within the gel. Considering the T1 and T2 relaxation times, as well as ADC values, we see stable values over 7 days with a small standard deviation. A higher variation was observed only in the MTR values (Figure 3). In the analysis by spectroscopy, no signals from substances other than water could be clearly detected (Figure 4A, Table 3).

### 3.2. Fibrin Glue

**Figure 5:**
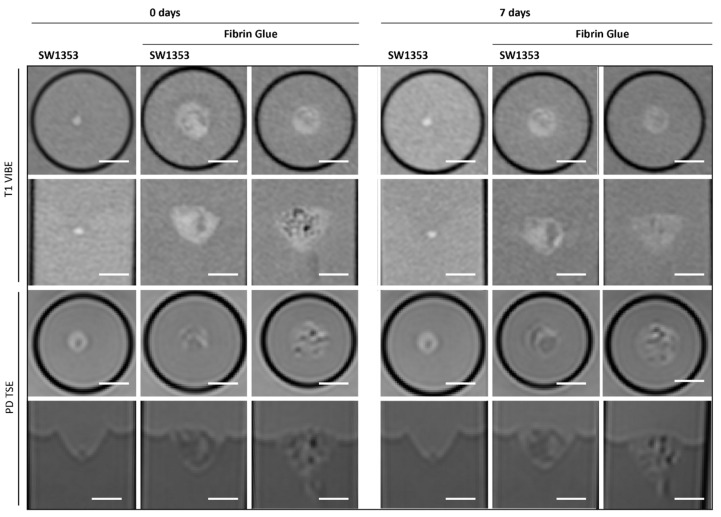
Cross-sectional (1st and 3rd row) and longitudinal axis (2nd and 4th row) images of tubes with SW1353 spheroids without and with fibrin glue and just fibrin glue. T1-weighted images (1st and 2nd row) were obtained using a spoiled gradient echo VIBE sequence. PD-weighted images were obtained using a TSE sequence. Scale bars represent 10 mm.

FG causes spatially extended signal enhancement in T1-weighted images. The presence of air inclusions, however, is an inevitable consequence of the application of FG, which can result in distinct local magnetic field inhomogeneities and signal extinctions in GRE images. Distinguishing between the spheroid clusters and the matrix on T1 and proton density imaging scans is therefore critical and no values for contrast could be obtained. It is important to note that the presence of air bubbles within the sample can introduce significant variability across repeated measurements (Figure 5). However, the temporal stability of FG was demonstrated in the present study, with no degradation observed over a period of seven days (Figure 3). Interestingly, FG displays the highest MTR value of all tested matrices (0.2398). The spectroscopic profile of FG can be characterized by a lack of clearly defined peaks, with the resulting spectrum being dominated by noise (Figure 4B, Table 3).

### 3.3. PEG PVA Hydrogel

**Figure 6:**
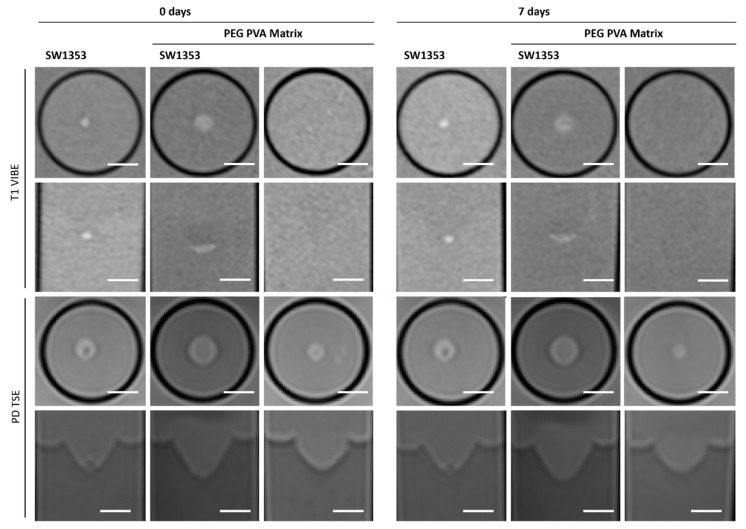
Cross-sectional (1st and 3rd row) and longitudinal axis (2nd and 4th row) images of tubes with SW1353 spheroids without and with PEG PVA hydrogel and just PEG PVA hydrogel. T1-weighted images (1st and 2nd row) were obtained using a spoiled gradient echo VIBE sequence. PD-weighted images were obtained using a TSE sequence. Scale bars represent 10 mm.

When examining images of PEG PVA hydrogel in MRI, a similar pattern emerges as for using Matrigel^®^: the matrix itself is barely recognizable, as it is indistinguishable from the signal of the medium, or even from the agar in T1-weighted imaging (Figure 6). This allows for clear, sharp-edged representation of the spheroids without interference. However, it was difficult to assess the position of the cell spheroid clusters within the hydrogel because areas with hydrogel are not clearly delineated from pure medium. Unlike Matrigel^®^, this condition persists even after one week and contrast remains stable in all image modalities (Table 3). The examination of PEG PVA hydrogel using MRS shows a signal at 1.12 ppm in addition to the water signal, with an amplitude of approximately 1.3% of the water signal. Two further minor signals (representing 0.26% and 0.09% of the water signal) are observed in the following sequence (Figure 4C). Fresh PEG PVA hydrogel demonstrates minimal variation in T1 and T2 relaxation times, as well as in ADC and MTR values, while these properties exhibit diminished stability after seven days (Figure 3, Table 3).

### 3.4. PEG Dex Hydrogel

**Figure 7:**
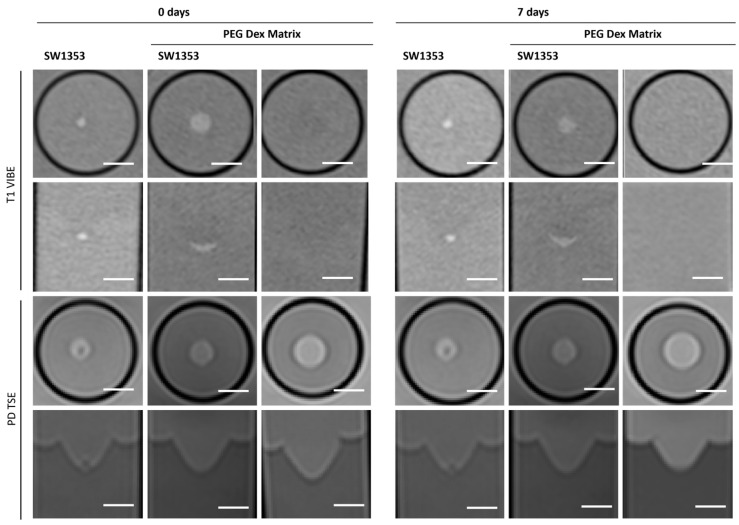
Cross-sectional (1st and 3rd row) and longitudinal axis (2nd and 4th row) images of tubes with SW1353 spheroids without and with PEG Dex hydrogel and just PEG Dex hydrogel. T1-weighted images (1st and 2nd row) were obtained using a spoiled gradient echo VIBE sequence. PD-weighted images were obtained using a TSE sequence. Scale bars represent 10 mm.

The replacement of the crosslinker from PVA to Dextran does not change the characteristics displayed in MRI. The hydrogel still does not emit its own signal, whereas the spheroids can be clearly visualized (Figure 7). Fresh PEG Dex hydrogel does not exhibit high variation in T1 and T2 relaxation times, as well as in ADC and MTR values, while these properties become less stable after 7 days (Figure 3). The spectroscopic signature exhibited a signal at 1.03 ppm below the water signal, with an intensity of 1.14%. No additional signals were detected (Figure 4D, Table 3).

### 3.5. PEG HA Hydrogel

**Figure 8:**
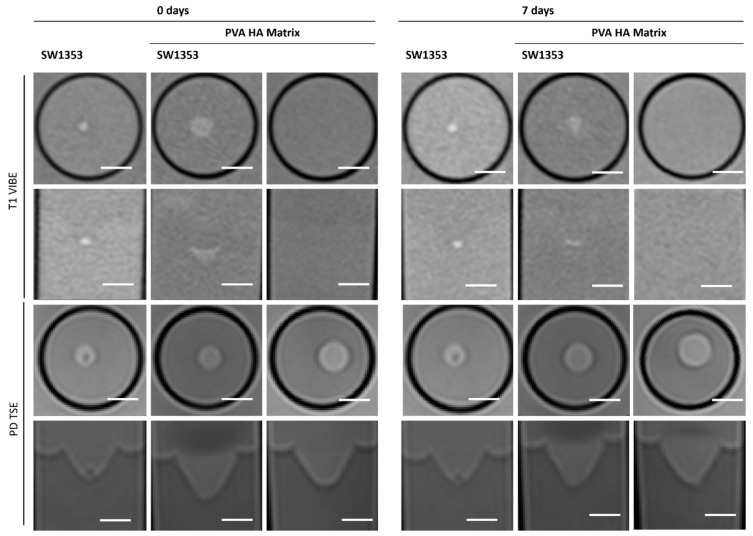
Cross-sectional (1st and 3rd row) and longitudinal axis (2nd and 4th row) images of tubes with SW1353 spheroids with and without PVA HA hydrogel and just PVA HA hydrogel. T1-weighted images (1st and 2nd row) were obtained using a spoiled gradient echo VIBE sequence. PD-weighted images were obtained using a TSE sequence. Scale bars represent 10 mm.

The PVA HA matrix itself is barely discernible from the signal of the surrounding medium, or even from the agar in T1-weighted images. As is the case with the other hydrogels, this one too facilitates the clear and sharp representation of the spheroids (Figure 8). It is notable that the optimal imaging conditions persist even after a period of one week with the utilization of the PEG PVA hydrogel (Figure 3). MRS merely revealed a noise pattern. PVA HA hydrogels have been observed to demonstrate minimal standard deviation in the immediate aftermath of the procedure. However, after a period of seven days, a substantial increase in standard deviation has been noted in the measurements taken. In contrast to the other two hydrogels containing PEG, no signals other than water were detected (Figure 4E; Table 3).

### 3.6. Polystyrene Insert

**Figure 9:**
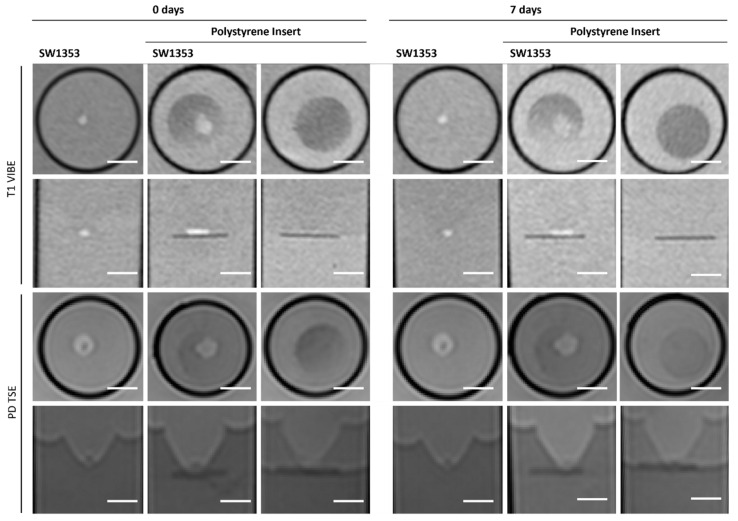
Cross-sectional (1st and 3rd row) and longitudinal axis (2nd and 4th row) images of tubes with SW1353 spheroids, native and on a polystyrene insert, and a polystyrene insert without cells. T1-weighted images (1st and 2nd row) were obtained using a spoiled gradient echo VIBE sequence. PD-weighted images were obtained using a TSE sequence. Scale bars represent 10 mm.

The polystyrene insert causes signal distinctions when placed inside the tube and is therefore clearly visible with negative contrast in both T1-weighted and proton density imaging. It is evident that the position of the spheroids can be clearly identified if they are located on the insert. However, the position of cell clusters within the mesh cannot be distinguished (Figure 9, Table 3). It is not possible to assess the disc quantitatively or via MRS due to the disc’s small dimensions (1.5 mm thickness).

To provide a comprehensive overview of all results and outcomes related to scaffold performance, we summarized key parameters affecting reproducibility and imaging quality, namely MRS compatibility, long-term culture stability, usability in handling, and cost-efficiency. These comparative findings are compiled in Table 4.

## 4. Discussion

The continuous advancement of magnetic resonance imaging (MRI) has significantly enhanced its applicability for the non-invasive assessment of geometrical distribution and composition of cell spheroid clusters, establishing its relevance in preclinical research as well as in vivo studies [39,40,41]. In order to provide the cells with an ideal environment, they are often cultivated and visualized in matrices [5,6]. For improved contrast and spatial distinction from the surrounding medium, cells are commonly labeled with iron-containing nanoparticles [42,43]. However, such labeling hinders the measurement of other physical properties of the spheroids. Therefore, a systematic investigation was undertaken to evaluate the feasibility and precision of visualizing unlabeled, native cell spheroids within various commonly used matrix systems.

Our results demonstrated pronounced differences in MRI signal intensity, relaxation behavior, and contrast profiles across the examined matrices and scaffolds, with MTR imaging displaying the strongest contrast. Quantitative analysis confirmed that these materials exhibit distinct magnetic and structural properties, which markedly influence the detectability and delineation of embedded cell aggregates. While none of them exhibited strong diamagnetic or paramagnetic characteristics, their signal-modifying properties varied vastly. These findings underscore that not all support matrices are equally suited for high-resolution MR imaging of three-dimensional cell constructs.

The MR characteristics of scaffolds and matrices are fundamentally determined by the water displacement and/or interaction of macromolecules with water in the medium. The concentration of the gel material and the number of cross-links per unit volume might influence MR-related features. Interactions of macromolecules with water often result in significantly shorter T2 relaxation times. In the case of high pore sizes, there is minimal restriction on the water molecules, which leads to their behavior being analogous to that of free water. Adhesive forces between the matrix surface and the water molecules might lead to bound water molecules with a very short T2, while magnetization transfer experiments can reveal exchange processes between free and bound water pools. In summary, while the overall water content primarily affects the overall MR signal intensity (proton density), the T2 relaxation time is a sensitive marker for the material’s micro-architecture, porosity, and surface interactions [44,45,46].

Among the materials tested, Matrigel^®^ remains one of the most commonly employed matrices in cell culture applications; however, it constitutes a comparatively costly option (Table 4) [15]. The localization and assessment of spheroids in MRI is possible without marked interferences if the cells were freshly embedded in Matrigel^®^ (the signal of which is nearly isointense to the surrounding media). Following a period of seven days, the spheroids no longer emit a sufficient signal to be localized within the matrix. Macroscopically, it was observed that the cells in the Matrigel^®^ migrate over the available surface area. The individual spheroids are too small for the available resolution achieved using 3T whole--body MRI. The mean T2 value for Matrigel^®^ was 736 ms, placing it in the intermediate position among the matrices examined. Given the minimal pore size of a few micrometers, it is plausible that nearly unrestricted Brownian motion was found with ADC values close to those in pure water [47]. Although Matrigel^®^ demonstrates temporal stability under static conditions, its use is limited to short-term imaging applications within this experimental configuration. This observation aligns with the findings of Mun et al. [48], who reported progressive loosening and degradation of the Matrigel^®^ matrix over time, accompanied by the appearance of dark granules after approximately two weeks in culture. Nevertheless, in experimental settings where spheroids are cultured and differentiated within Matrigel^®^, the matrix was typically renewed at regular intervals; thus, it remained suitable for most short- and medium-term applications [49,50].

In contrast, FG was found to be unsuitable as a matrix material for applications involving MR-based evaluation. The handling and application procedure present inherent technical challenges, as even minimal air inclusions give rise to pronounced susceptibility artifacts. Interestingly, with a pore size of 100–11,000 nm [51], FG displayed the lowest T2 value comparable to that of 1% (*w*/*v*) agar. In addition, positive magnetization transfer effects indicate the presence of a bound water pool in FG, exchanging magnetization with free water molecules. Moreover, even under optimized conditions, reliable assessment of spheroids remains unattainable due to the insufficient intrinsic contrast between the fibrin matrix and the cellular aggregates, thereby precluding accurate visualization and quantitative analysis. This limitation is particularly noteworthy given the widespread clinical use of FG as a carrier for mesenchymal stem cells in cartilage repair, where postoperative monitoring is commonly performed using MRI [52,53]. Consequently, MR-based assessment in such cases is effectively confined to the broader regeneration zone surrounding the implanted FG.

All three synthetic hydrogels exhibited analogous characteristics in MRI with the exception of PEG PVA, which displays shorter T2 values. The embedded cell spheroid clusters could be clearly delineated, and their MR signal was not attenuated by the surrounding gel matrix. In contrast to Matrigel^®^, this property retains its integrity for a period of seven days. Beyond this time point, however, quantitative MRI parameters such as signal intensity and relaxation values showed markedly increased variability, as reflected by elevated standard deviations relative to freshly prepared gels. This effect appeared independent of the polymer composition, occurring in both PVA- and dextran-based hydrogels. Notably, the manufacturer’s documentation provides no specification regarding the shelf life or long-term structural stability of the cast gels. Hydrogel degradation has been shown to induce alterations in mechanics and swelling over time. Such changes have been demonstrated to influence key cellular processes, including motility, spreading, and traction force generation [54]. Furthermore, matrix-metalloproteinases (MMPS) secreted by cells are capable of degrading various extracellular matrix (ECM) proteins commonly incorporated into hydrogels, such as fibronectin, laminin, collagen, or gelatin [55]. Fully synthetic hydrogels are less prone to degradation. Nonetheless, recent studies have increasingly focused on the controlled modification of synthetic hydrogels to render them responsive to cellular remodeling processes, for instance, by enabling cell-mediated degradation and migration [56,57]. It should be noted that neither Matrigel^®^ nor the three tested hydrogels allow a clear delineation between the matrix and surrounding media. This has the potential to cause issues in situations where precise localization is paramount, and it is therefore advisable to consider this aspect in advance.

In addition to hydrogel-based systems, alternative synthetic scaffolds such as polystyrene inserts can also be employed within this experimental framework, though with certain limitations. Cells located on the surface of the scaffold can be clearly visualized and quantitatively analyzed; however, cells embedded within the plate material cannot be distinguished from the surrounding synthetic matrix. Consequently, the obtained measurements represent only a fraction of the total cellular population. Despite this constraint, polystyrene inserts constitute a practical and cost-effective alternative (i.e., €1.19 per piece) to other scaffold materials evaluated in this study (Table 4).

The spectroscopic profiles of the evaluated matrices offer valuable insights into potential peak interferences that may affect the accuracy of cell cluster measurements. Among these materials, the HA PVA hydrogel provides an optimal environment for spectroscopic analysis, as it exhibits no detectable signal other than the intrinsic water peak, thereby enabling precise and interference-free quantification. Consequently, signal interference is not anticipated when employing this matrix. Furthermore, Matrigel^®^ and FG exhibited no peaks other than water in the measurements conducted. However, the noise is marginally more pronounced in these two matrices. Nevertheless, this is not at a level that would obscure any significant peaks. Hydrogels containing PEG exhibited a distinct peak at around 3.9 ppm. Investigations of PEG using high-field spectroscopy also detected signals within this range. Therefore, it can be deduced that the measured signal is likely to correspond to the -CH_2_CH_2_O groups [58,59]. Prior to the utilization of these hydrogels, it is imperative to consider whether signals within a comparable range are anticipated. In the event that this is the case, it is recommended that a different gel is used in order to avoid overlaps or confusion.

As a consequence, the selection of matrices and scaffolds for spheroid cultures intended for MR-based monitoring should be thoroughly validated prior to experimental use, as not all materials provide the necessary contrast, stability, or signal integrity for reliable imaging. Moreover, both the duration of the experiment and the intended observation period must be carefully considered, since temporal changes in matrix properties can substantially affect imaging quality and data interpretation. Collectively, these findings underscore the importance of a deliberate and material-specific approach when designing MR-compatible 3D cell culture systems.

This work evaluates the suitability and properties of various matrices and scaffolds for visualization in 3 Tesla MRI. A range of different matrices have been analyzed, encompassing synthetic, semi-synthetic and natural varieties. However, it should be noted that there are a significant number of other matrices that have not been included in this study. Furthermore, the spatial resolution at 3 Tesla is limited, thus preventing a more accurate evaluation. The utilization of high-field systems would facilitate the acquisition of more detailed results in this context and very-high-field MRI systems with dedicated RF microcoils would even allow assessment of single spheroids in regard of size and morphology.

## 5. Conclusions

This study demonstrates that the choice of matrix or scaffold critically determines the feasibility and quality of MR-based assessments of three-dimensional cell spheroids. While natural matrices such as Matrigel^®^ enable short-term visualization, their structural instability limits long-term imaging. In contrast, synthetic hydrogels, particularly HA PVA, provide stable, interference-free conditions suitable for extended spectroscopic and imaging analyses. HA PVA fulfills all necessary criteria for successful imaging and is therefore the most promising scaffold in our opinion. FG, despite its clinical relevance, and polystyrene inserts present notable limitations due to imaging artifacts and restricted accessibility. Collectively, these findings highlight the necessity of material-specific validation and careful consideration of experimental duration when designing MR-compatible 3D culture systems.

## Figures and Tables

**Table 1:** Imaging protocol.

	PD/T2-w	T1-w	T1-Map	T2-Map	DWI	MTR
**Sequence name**	2D TSE	3D VIBE	3D VFA VIBE	2D CPMG	RESOLVE	3D GRE
Echo train length	18	1	1	32	1	1
TR (ms)	3000	6.8	9.2	3000	3000	25
TE (ms)	12/160	2.74	2.71	10–320	47	3.18
Flip angle (deg)	90–180n	10	2/8/15	90–180n	180	10
BW (Hz/Px)	191	190	190	235	744	190
Matrix	120 × 160, 15 slices	173 × 256 × 128	120 × 160 × 30	120 × 160 single slice	120 × 160 single slice	120 × 160 × 30
FOV (mm^2^/mm^3^)	120 × 160 × 16	116 × 154 × 38	120 × 1600 × 15	120 × 160 × 2	120 × 160 × 3	120 × 160 × 15
Voxel size (mm^3^)	1.0 × 1.0 × 1.0	0.6 × 0.6 × 0.6	1.0 × 1.0 × 1.0	1.0 × 1.0 × 2.0	0.5 × 0.5 × 3.0	0.5 × 0.5 × 0.5
Scan time (min:s)	3:41	4:28	5:37	6:05	6:53	4:48 × 2

**Table 2:** Spectroscopy protocol.

	MRS
**Sequence Name**	STEAM
TR (ms)	4000
TE (ms)	5.4
Voxel size (mm^3^)	3 × 3 × 3
Bandwidth	1400 Hz
nAcq	8 (without water suppression), 128 (with water suppression), 2 preparation scans

**Table 3:** Signal-to-noise ratio (SNR) of proton-density-weighted images measured from Matrigel^®^, PEG PVA hydrogel, PEG Dex hydrogel, PEG HA hydrogel, fibrin glue, agar, and media. Acquired mean values and standard deviations for T1, T2, ADC, and MTR values and their respective contrast with spheroids (*n* = 3) at 0 and 7 days after casting. Measurements were obtained from three representative defined regions of interest (ROIs).

		Matrigel		PEG PVA Hydrogel		PEG Dex Hydrogel		PEG HA Hydrogel		Fibrin Glue		Agar		Media	
**SNR (PD)**		127		103		116		108		125		104		119	
		Mean	SD	Mean	SD	Mean	SD	Mean	SD	Mean	SD	Mean	SD	Mean	SD
day 0	T1 [ms]	2779	191	2856	44	3098	166	2977	156	2441	475	2684	528	2929	85
	Contrast in T1	30%		24%		23%		20%		n.d. *		23%		24%	
	T2 [ms]	736	117	488	196	966	119	1078	238	166	25	121	6	1534	12
	Contrast in T2	−17%		−1%		−27%		−32%		n.d. *		−3%		−35%	
	ADC [10^−6^ mm^2/s^]	2077	188	2106	76	2147	64	2071	24	1975	14	2147	110	2317	103
	Contrast in ADC	−7%		−19%		−61%		−70%		n.d. *		−8%		−15%	
	MTR	0.0765	0.0567	0.0222	0.0069	0.0332	0.0183	0.0316	0.0070	0.2398	0.0501	0.1603	0.0402	0.0210	0.0041
	Contrast in MTR	60%		84%		90%		82%		n.d. *		−2%		86%	
day 7	T1 [ms]	2675	83	3292	205	3048	284	3002	124	2710	638	2810	6	2923	101
	Contrast in T1	3%		40%		15%		20%		n.d. *		3%		3%	
	T2 [ms]	807	190	718	416	826	399	797	303	267	147	203	180	1609	15
	Contrast in T2	−2%		−1%		−4%		−18%		n.d. *		19%		−3%	
	ADC [10^−6^ mm^2/s^]	2116	89	1858	262	1870	265	1890	327	1997	104	2149	102	2183	44
	Contrast in ADC	−1%		−20%		−10%		−40%		n.d. *		0		−6%	
	MTR	0.0200	0.0066	0.0654	0.0613	0.0537	0.0676	0.0742	0.0505	0.1779	0.0036	0.1316	0.0113	0.0437	0.0209
	Contrast in MTR	54%		53%		69%		63%		n.d. *		−2%		−82%	

n.d. * = not determined as FG and spheroids were not discernible.

**Table 4:** Summary of key properties of the scaffold materials evaluated, including MRI/MRS compatibility, culture stability, cost, and usability.

	Matrigel^®^	PEG PVA Hydrogel	HA PVA Hydrogel	PEG Dex Hydrogel	Fibrin Glue	Poly- Styrene Insert
**MRI Susceptibility**	Isointense with media	Isointense with media	Isointense with media	Isointense with media	Signal- enhancing	Signal depleting
Discernibility of cells	+/−	+	+	+	−	+
Stability in long-term culture	+	+/−	+/−	+/−	+	+
Artefact generation	−	−	−	−	Artefact generation through air inclusion	−
Approx. Cost	− EUR 48/mL	+ EUR 21/mL	− EUR 50/mL	+ EUR 23/mL	− EUR 52/mL	+ EUR 1.19 per plate
MRS Compatibility	−	+	+	+	+	−
Usability	+	+	+	+	−	−

## Data Availability

The original contributions presented in the study are included in the article, further inquiries can be directed to the corresponding author.

## Abbreviations

The following abbreviations are used in this manuscript:

ADC	apparent diffusion coefficient
CPMG	Carr–Purcell–Meiboom–Gill
Dex	dextran
DWI-EPI	diffusion-weighted echo planar imaging
ECM	extracellular matrix
EHS	Elbrecht–Holm–Swarm
FG	fibrin glue
HA	hyaluronic acid
MRI	magnetic resonance imaging
MRS	magnetic resonance spectroscopy
MTR	magnetization transfer ratios
PD	proton density
PEG	polyethylene glycol
PS	polystyrene
PVA	polyvinyl alcohol
SNR	signal-to-noise ratio
STEAM	stimulated echo acquisition mode
T	Tesla
TSE	turbo spin echo
VFA	variable flip angle
VIBE	volumetric interpolated breath-hold examination
